# Contrasting Community Assembly Mechanisms Underlie Similar Biogeographic Patterns of Surface Microbiota in the Tropical North Pacific Ocean

**DOI:** 10.1128/spectrum.00798-21

**Published:** 2022-01-12

**Authors:** Jie Kong, Lei Wang, Cai Lin, Fangfang Kuang, Xiwu Zhou, Edward A. Laws, Ping Sun, Hao Huang, Bangqin Huang

**Affiliations:** a State Key Laboratory of Marine Environmental Science, Fujian Provincial Key Laboratory for Coastal Ecology and Environmental Studies, Xiamen University, Xiamen, Fujian, China; b Southern Marine Science and Engineering Guangdong Laboratory (Zhuhai), Zhuhai, Guangdong, China; c Third Institute of Oceanography, Ministry of Natural Resources, Xiamen, Fujian, China; d Department of Environmental Sciences, College of the Coast and Environment, Louisiana State University, Baton Rouge, Louisiana, USA; Nanyang Technological University

**Keywords:** bacteria, alpha diversity, beta diversity, distribution patterns, microeukaryotes, tropical North Pacific Ocean

## Abstract

Marine microbiota are critical components of global biogeochemical cycles. However, the biogeographic patterns and ecological processes that structure them remain poorly understood, especially in the oligotrophic ocean. In this study, we used high-throughput sequencing of 16S and 18S rRNA genes to investigate the distribution patterns of bacterial and microeukaryotic communities and their assembly mechanisms in the surface waters of the tropical North Pacific Ocean. The fact that both the bacterial and the microeukaryotic communities showed similar distribution patterns (i.e., similar distance-decay patterns) and were clustered according to their geographic origin (i.e., the western tropical North Pacific and central tropical North Pacific) suggested that there was a significant biogeographic pattern of microbiota in the North Pacific Ocean. Indices of alpha diversity such as species richness, phylogenetic diversity, and the Shannon diversity index also differed significantly between regions. The correlations were generally similar between spatial and environmental variables and the alpha and beta diversities of bacteria and microeukaryotes across the entire region. The relative importance of ecological processes differed between bacteria and microeukaryotes: ecological drift was the principal mechanism that accounted for the structure of bacterial communities; heterogeneous selection, dispersal limitation, and ecological drift collectively explained much of the turnover of the microeukaryote communities.

**IMPORTANCE** Bacteria and microeukaryotes are extremely diverse groups in the ocean, where they regulate elemental cycling and energy flow. Studies of marine microbial ecology have benefited greatly from the rapid progress that has been made in genomic sequencing and theoretical microbial ecology. However, the spatial distribution of marine bacteria and microeukaryotes and the nature of the assembly mechanisms that determine their distribution patterns in oligotrophic marine waters are poorly understood. In this study, we used high-throughput sequencing methods to identify the distribution patterns and ecological processes of bacteria and microeukaryotes in an oligotrophic, tropical ocean. Our study showed that contrasting community assembly mechanisms underlaid similar biogeographic patterns of surface bacterial and microeukaryotic communities in the tropical North Pacific Ocean.

## INTRODUCTION

Marine microbiota, including prokaryotes (i.e., bacteria and archaea) and microeukaryotes (i.e., fungi and protists), are among the critical components of global biogeochemical cycles (1, 2). Bacteria and microeukaryotes constitute most of the marine microbiota in oligotrophic ocean surface waters, where they regulate elemental cycling and energy flow (3). Although bacteria and microeukaryotes differ with respect to cellular structure, cell size, metabolic diversity, and evolutionary strategies (4), they live in the same habitat and are directly and indirectly linked through biogeochemical cycles and food web interactions (5, 6). A comprehensive understanding of the ecology of marine bacteria and microeukaryotes, therefore, requires that they be investigated simultaneously (7–9).

One central goal of marine microbial ecology is to elucidate the spatiotemporal distribution patterns of marine microbiota (i.e., their biogeography) and to identify the mechanisms responsible for those patterns (10). Studies of marine microbial biogeography have benefited greatly from the rapid progress that has been made in genomic sequencing and theoretical microbial ecology (11). Among the various spatial distribution patterns (e.g., latitudinal diversity patterns and taxa-area relationships), distance-decay patterns have frequently been reported based on studies of communities of bacteria and microeukaryotes in a variety of aquatic habitats, including rivers (12, 13), lakes (9, 14), intertidal zones (15), coastal ocean (8, 16), and open ocean (16, 17). However, there have been few studies of microbial biogeographic patterns in oligotrophic marine waters, especially at the trans-basin scale (17).

Knowledge of the mechanisms that shape microbial biogeographic patterns is necessary to understand the structure of microbial communities and to exercise best practices in the stewardship of marine resources (11, 18). According to the conceptual synthesis of Vellend (19), the ecological processes that shape the patterns of communities include selection (reproductive success influenced by biotic and abiotic pressures), dispersal (spatial movements of individuals), ecological drift (random fluctuations of population sizes due to stochastic events) and speciation (evolution of new species). Stegen et al. (20) have developed an analytical framework to estimate the relative influences of selection (heterogeneous and homogenizing selection), dispersal (dispersal limitation and homogenizing dispersal), and ecological drift. The relative importance of different assembly processes has been found to differ among taxonomic groups and habitats (7, 9, 15, 20, 21). Logares et al. (9), for example, have found that habitat diversification can result in contrasting assembly mechanisms: in the lakes of East Antarctica, bacterial communities are shaped mainly by selection while microeukaryotic communities are structured mainly by ecological drift. In the tropical and subtropical surface ocean, the biogeographies of prokaryotes and picoeukaryotes are shaped by different mechanisms. Dispersal limitation predominantly shapes picoeukaryotic communities, whereas dispersal, selection, and ecological drift collectively structure prokaryotic communities (7). The underlying assembly mechanisms that shape bacterial and microeukaryotic communities in the oligotrophic surface ocean are unclear.

In this study, we investigated the distribution patterns and assembly mechanisms of bacterial and microeukaryotic communities in the oligotrophic surface waters of the tropical North Pacific Ocean (TNPO). Our specific goals were to answer the following key questions. (i) Do bacteria and microeukaryotes exhibit similar or different distribution patterns? (ii) What are the factors that determine the diversities of bacterial and microeukaryotic communities? (iii) What is the relative importance of selection, dispersal, and ecological drift in determining the distribution of bacteria and microeukaryotes?

## RESULTS

### Environmental characteristics.

**FIG 1:**
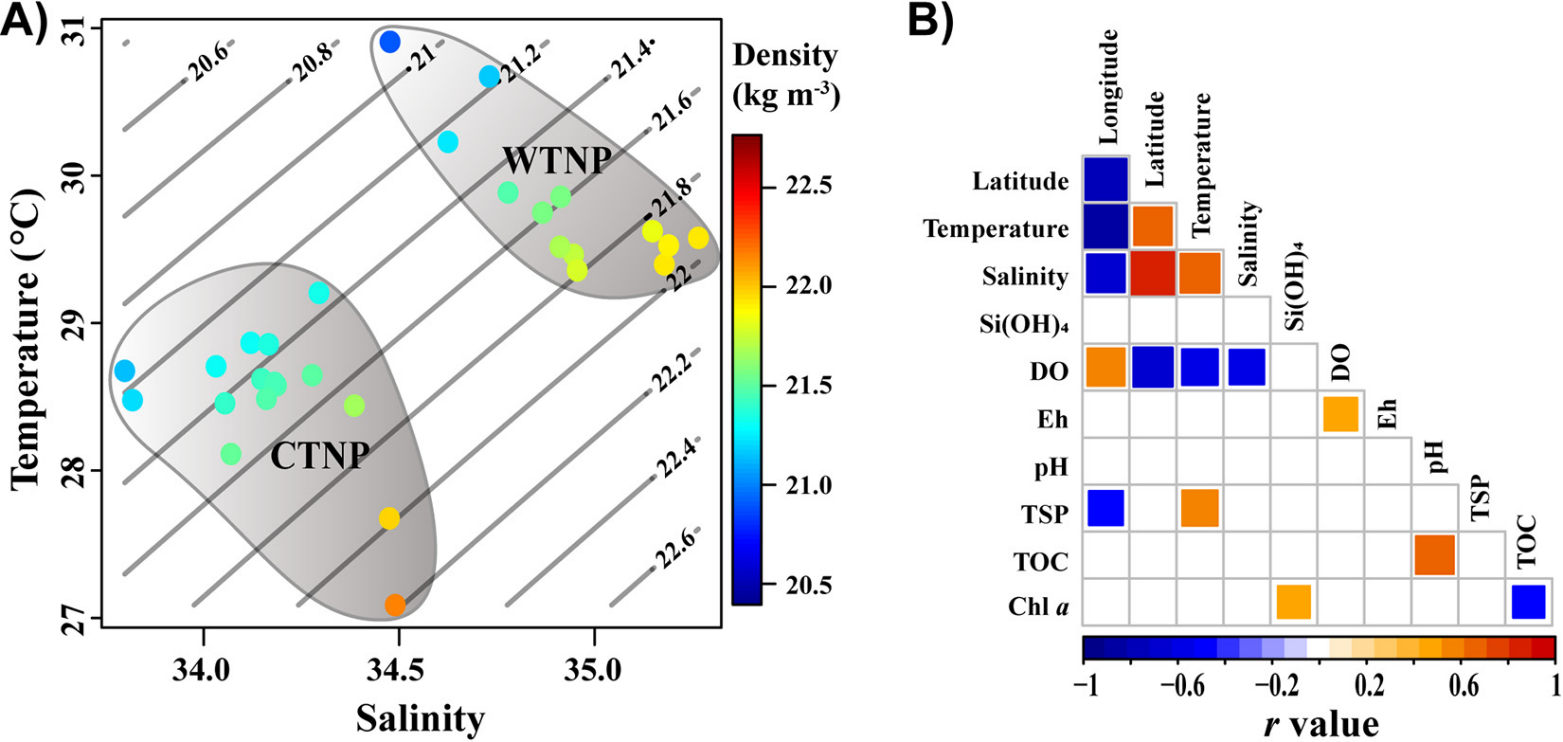
(A) Temperature-salinity diagram of surface water during our sampling. (B) Spearman correlation matrix showing the results of correlation tests among spatial and environmental variables. The *P* values were adjusted by the “fdr” method. Only significant correlations (adjusted *P* < 0.05) are shown in the Spearman correlation matrix. WTNP, western tropical North Pacific Ocean (13 stations); CTNP, central tropical North Pacific Ocean (16 stations); DO, dissolved oxygen; Eh, reduction potential; TSP, total suspended particulates; TOC, total organic carbon.

Table S1 in the supplemental material shows the values of the environmental variables. The temperature, salinity, dissolved oxygen (DO), and oxidation-reduction potential (Eh) were lower in the western tropical North Pacific Ocean (WTNP) than in the central tropical North Pacific Ocean (CTNP; Fig. 1A). The distinction between the WTNP and CTNP stations was further confirmed by analysis of similarity (ANOSIM; R = 0.526, P < 0.001) (Fig. S1). The pH values and concentrations of total suspended particulates (TSP), total organic carbon (TOC), Si(OH)_4_, and chlorophyll a (Chl a) were not significantly different between the WTNP and CTNP (Fig. S2).

Many significant correlations were found between the spatial and environmental variables (Fig. 1B). These patterns differed, however, when the WTNP and CTNP were considered independently (Fig. S3). Moreover, the fact that pairwise environmental variances were significantly but weakly correlated with increasing geographic distances (*R*^2^_adj_ = 0.212, *P* < 0.001; Fig. S4A) indicated that environmental factors were associated with distance-decay patterns. In contrast, environmental factors showed no distance-decay pattern within the WTNP and CTNP (Fig. S4B).

### Alpha diversity of microbiota.

Rarefaction curves showed that all amplicon samples of bacterial and microeukaryotic communities were almost saturated with respect to the number of sequences (Fig. S5). With 97% similarity, 2,182 and 4,760 operational taxonomic units (OTUs) were recovered for bacterial and microeukaryotic communities, respectively, when nonbacterial/non-microeukaryotic OTUs were filtered out and OTU tables were randomly rarefied to 95,250 sequences. Table S2 shows the detailed alpha diversity indices. The number of OTUs is a metric of richness, and the samples contained 369 to 1,150 bacterial OTUs (mean: 665 OTUs) and 1,292 to 2,148 microeukaryotic OTUs (1,683 OTUs; Table S2).

**FIG 2:**
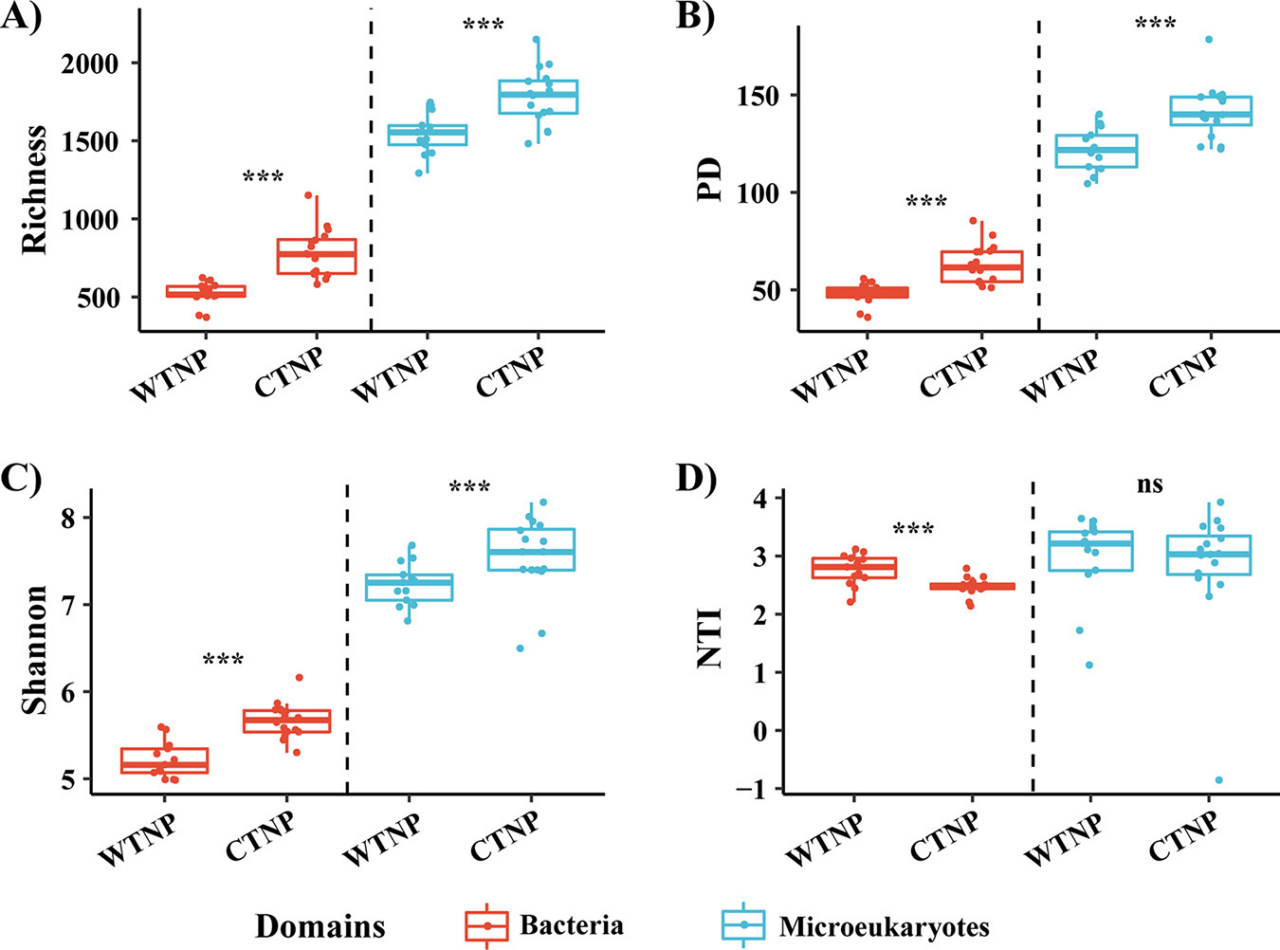
Boxplot comparing mean values of alpha diversity indices. (A) Species richness; (B) PD; (C) Shannon; (D) NTI between regions. Vertical dashed lines separate comparisons. Mean value comparisons were performed using a Wilcoxon test. ***, significant differences; ns, no significant differences.

All the nearest taxon index (NTI) values for bacteria were significantly different from zero, and 30 of the 32 NTI values for microeukaryotes were significantly greater than zero (Table S2). Both taxonomic communities, therefore, were phylogenetically clustered (relative to random communities). For both bacterial and microeukaryotic communities, the species richness, Faith’s phylogenetic diversity (PD), and Shannon indices were significantly higher in the CTNP than in the WTNP (Fig. 2A–C). The implication is that the central and western regions of the TNPO are associated with distinctly different alpha diversity-patterns. However, the NTI indices of the bacteria were higher in the WTNP than in the CTNP, and there was no significant difference between the NTI indices of microeukaryotes in the WTNP and CTNP (Fig. 2D).

In the entire region, richness and PD indices were significantly correlated between domains (i.e., bacteria and microeukaryotes) (*P* < 0.001; *R*^2^ = 0.448 and 0.653, respectively), whereas no significant correlation was found in the cases of the Shannon and NTI indices (*P > *0.05) for bacteria and microeukaryotes (Fig. S6). When the WTNP and CTNP were considered separately, only the PD indices of the bacterial and microeukaryotic communities were significantly correlated (Fig. S7).

### Distribution patterns of microbiota.

**FIG 3:**
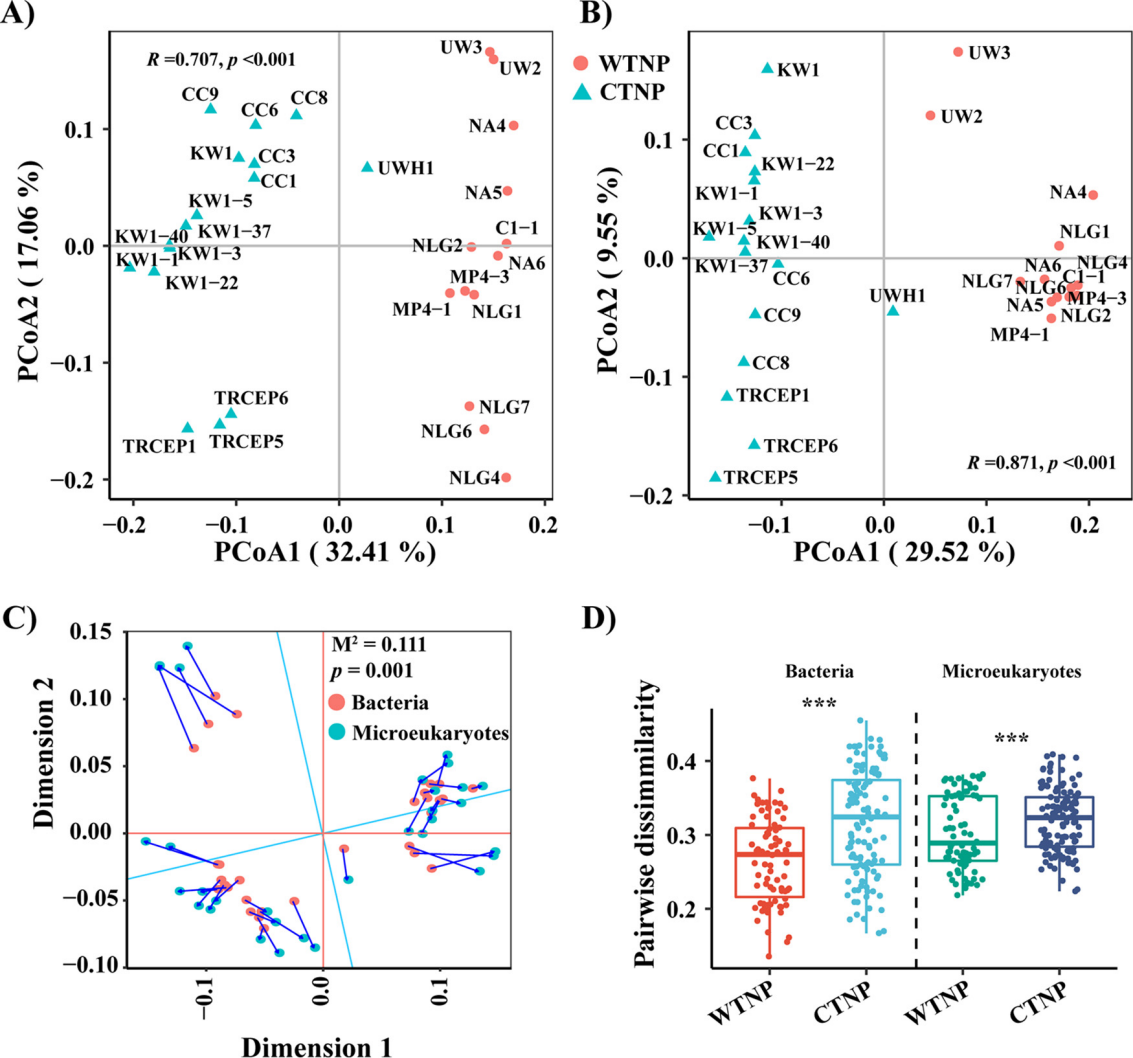
PCoA showing the distribution patterns of (panel A) bacteria and (panel B) microeukaryotes based on Bray-Curtis distances. (C) Procrustes analysis to evaluate the agreement of distribution patterns of bacterial and microeukaryotic communities based on principal coordinates analysis, with statistical significance measured by Monte Carlo test. (D) Boxplot comparing mean values of pairwise dissimilarities between regions (WTNP versus CTNP). In panels A and B, the *R* and *P* statistics are the results of ANOSIM analysis with 9,999 permutations that were used to further statistically assess between-group differences. (C) Blue lines between pairs of points indicate that the two connected points represent the same sample. (D) The vertical dashed line separates comparisons, and significant differences are marked with *** (Wilcoxon test).

Bacterial and microeukaryotic communities clustered according to their geographic origin, i.e., the WTNP and CTNP (Fig. 3A–B). These clusters were further verified using ANOSIM analysis; for bacteria and microeukaryotes, *R *=* *0.707 and 0.871, respectively (Fig. 3A–B). Procrustes analysis revealed that bacterial communities showed distribution patterns similar to those of microeukaryotes (*M*^2^ = 0.111, *P = *0.001; Fig. 3C). Furthermore, the pairwise beta diversities of the two community types were significantly correlated for the entire study region (*R*^2^_adj_ = 0.593, *P* < 0.001) as well as for the WTNP (*R*^2^_adj_ = 0.717, *P < *0.001) and CTNP (*R*^2^_adj_ = 0.414, *P < *0.001; Fig. S8). Pairwise dissimilarities of the CTNP microbial communities were significantly higher than those of the WTNP (bacteria: W = 2739, *P < *0.001; microeukaryotes: W = 3809, *P* = 0.027; Fig. 3D).

**FIG 4:**
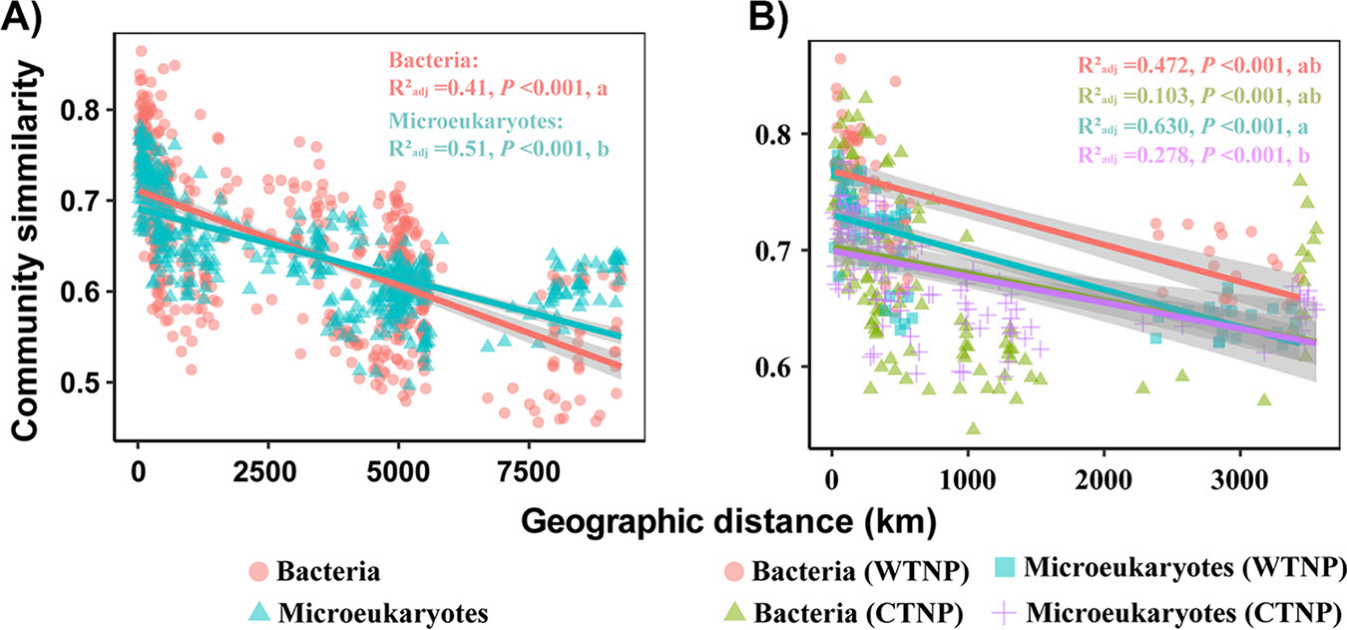
Distance-decay patterns of bacteria and microeukaryotes for the TNPO (panel A) and the WNTP and CNTP (panel B). Community dissimilarity is based on the Bray-Curtis distance. Solid line shows the best linear fit. Lines with significantly different slopes are labeled with different lowercase letters.

The community similarities of both bacteria and microeukaryotes decreased with increasing geographical distances between stations. In other words, the similarities exhibited distance-decay patterns for the entire study region (Fig. 4A) as well as for the WTNP and CTNP (Fig. 4B). It should be noted that the slopes of the distance-decay patterns differed between domains and regions. For example, the fact that the slopes of distance-decay patterns were significantly steeper for bacteria than for microeukaryotes indicated that the patterns for bacteria exhibited a stronger distance-decay relationship (Fig. 4A). A significant difference between slopes of the distance-decay patterns between the WTNP and CTNP was observed only for microeukaryotes (Fig. 4B).

### Factors influencing the microbiota.

**FIG 5:**
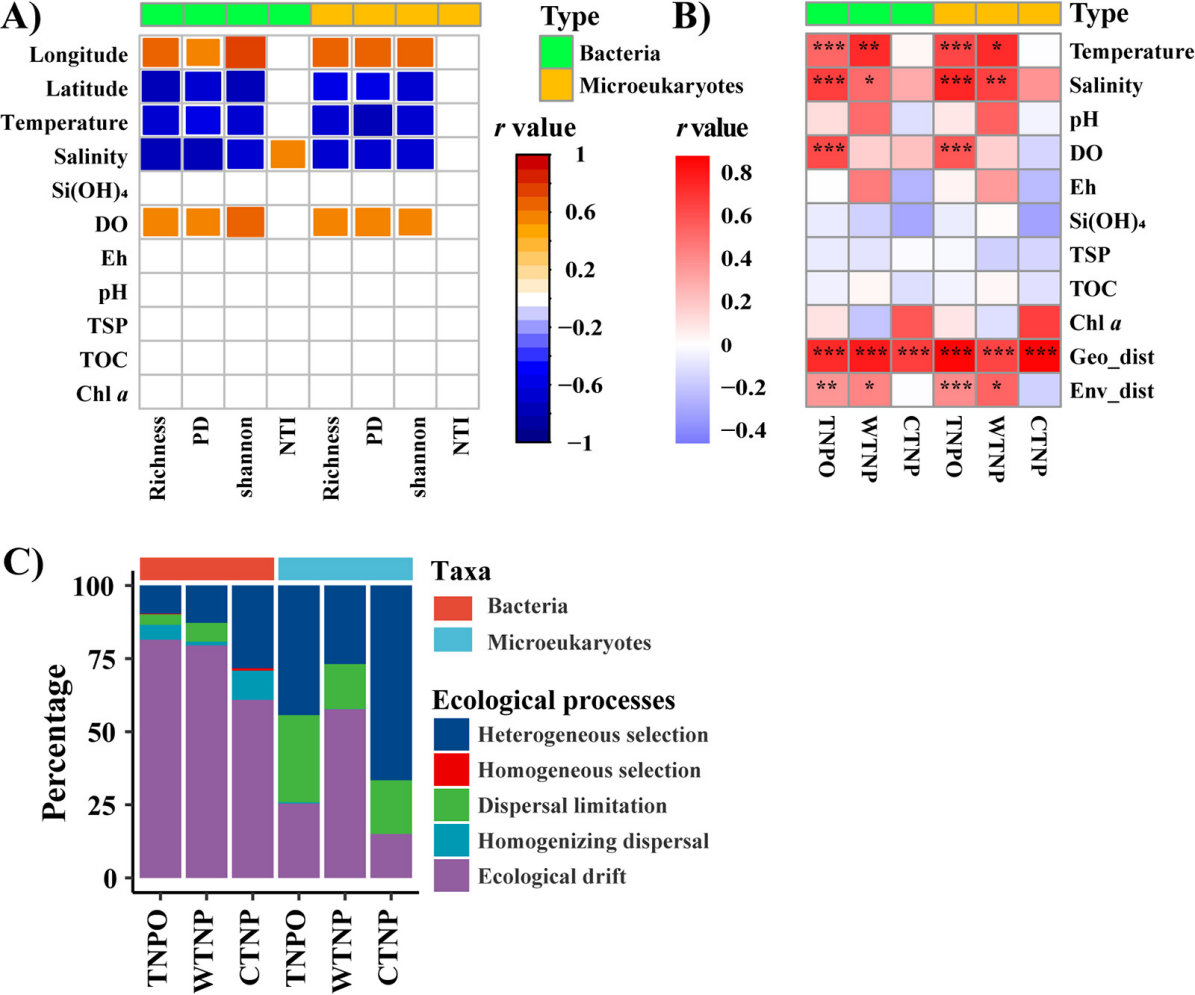
The factors correlating with alpha- (panel A) and beta- (panel B) diversities of bacteria and microeukaryotes. Quantification of the relative contributions of the ecological processes shaping bacterial and microeukaryotic communities (panel C). Heatmaps showing the results of Spearman rank correlation tests and Mantel tests for (panel A) alpha diversity and (panel B) beta diversities of both domains. Only significant correlations (adjusted *P* < 0.05) are shown in the heatmaps. In panel B, *P* < 0.05, <0.01, and <0.001 are indicated by *, **, and ***, respectively. Geodist, pairwise geographical distances; Envdist, Euclidean distance of all measured environmental variables.

Correlations between alpha diversity indices and measured environmental and spatial variables generally exhibited similar patterns for bacterial and microeukaryotic communities across the entire study region. For example, location (longitude and latitude), temperature, salinity, and DO concentration were significantly correlated with microbial indices, with the exception of the NTI. Salinity was positively correlated with NTI in bacteria (Fig. 5A). Correlations between alpha diversity indices and measured environmental and spatial variables differed between domains and regions (i.e., WTNP and CTNP; Fig. S9).

Mantel tests showed that the variations of both bacterial and microeukaryotic communities were correlated with the same factors, both in the entire tropical North Pacific and in the WTNP and CTNP (Fig. 5B). For the entire study region, environmental factors such as temperature, salinity, and DO concentrations were significantly related to both bacterial and microeukaryotic community variances, while temperature and salinity were significantly correlated with community variances of both domains in the WTNP. In contrast, no measured environmental variables were significantly correlated with either bacterial or microeukaryotic community variances in the CTNP (Fig. 5B). In addition, beta-diversity dissimilarities of both bacteria and microeukaryotes increased significantly with increases of geographic or environmental differences for the entire study region as well as the WTNP and CTNP, except for environmental distance in the CTNP (Fig. 5B).

### Assembly mechanisms of microbial communities.

The phylogenetic model analysis revealed that the relative contributions of ecological processes differed between domains and regions (Fig. 5C). Ecological drift and homogenizing dispersal generally had larger impacts on bacteria than microeukaryotes, whereas heterogeneous selection and dispersal limitation had larger impacts on microeukaryotes than bacteria. In addition, homogeneous selection contributed a negligible proportion of the turnover of both domains in all cases. Specifically, ecological drift, followed by heterogeneous selection, were the ecological processes that had the greatest impact on bacterial communities, whereas the processes involved in assembly of microeukaryotic communities were collectively determined by heterogeneous selection, dispersal limitation, and ecological drift. The relative contributions of these mechanisms differed between groups (Fig. 5C).

## DISCUSSION

### Alpha-diversity patterns of microbiota.

The observed alpha diversity indices could be influenced by many methodological factors such as primer choices, sequencing depths, bioinformatics processing pipelines, and variable numbers of gene copies (22, 23). In this study, many strategies were used to minimize such methodological uncertainties. For example, DNAs extracted from the same filter were used for bacterial 16S and eukaryotic 18S rRNA gene sequencing, and the same pipeline was used to analyze bacterial and eukaryotic gene sequences.

The fact that the richness, PD, and Shannon indices of both bacterial and microeukaryotic communities were higher in the CTNP than in the WTNP (Fig. 2) indicated that regions of the TNPO could harbor significantly distinct alpha-diversity patterns. A recent study has shown that richness of bacteria and microeukaryotes vary in different oceanic provinces in the South Pacific Ocean, where environmental variables such as temperature, salinity, and DO differ among oceanic provinces (24). Our results revealed that the WTNP and CTNP have distinct physiochemical properties (Fig. 1A, Fig. S1 and S2) that might account for the different alpha diversity indices of the microbial communities in those areas. It should be noted that not all the patterns of the alpha indices were similar between bacteria and microeukaryotes, although they harbored the same circumstances and were directly and indirectly linked for the entire TNPO as well as for the CTNP and WTNP (Fig. S6 and S7). As reviewed by Keeling and Campo (4), there are intrinsic differences between the attributes (for example, cellular structure, cell size, metabolic diversity, and evolutionary strategies) of bacterial and microeukaryotic communities that likely contribute to the differences of their alpha-diversity patterns. The differences between the alpha-diversity patterns of the bacteria and microeukaryotes might indicate that those two domains have been shaped by different assembly mechanisms in the TNPO. Because the patterns revealed by different indices varied, achieving a comprehensive understanding of microbial communities will require that different types of indices be included in future studies.

The correlations between spatial and environmental variables and alpha diversity indices were similar across the TNPO for bacteria and microeukaryotes, but they differed in the WTNP and CTNP (Fig. 5A and Fig. S9). Li et al. (25) have reported that all measured variables were uncorrelated with species richness, Chao 1 indices, and Shannon indices of bacteria in the Northwestern Pacific Ocean. In the subtropical Northwest Pacific Ocean, only the PD index of bacteria has been reported to be positively correlated with concentrations of nitrite + nitrate (17). Raes et al. (24) have shown that correlations between biogeochemical parameters and the species richness of bacteria and microeukaryotes differ between oceanic provinces. Because environmental variables such as temperature, salinity, DO, and Eh differed significantly between the WTNP and CTNP (Fig. S1), it would be reasonable to expect higher heterogeneity of those variables in the TNPO than in the WTNP and CTNP separately. The greater heterogeneity of environmental variables within the TNPO likely impacts the composition of bacterial and microeukaryotic communities, and therefore might help to explain the differences between the correlations observed between environmental variables and diversity in the TNPO compared to the WTNP and CTNP separately (Fig. 5A and Fig. S2 and S9).

### Beta-diversity patterns of microbiota.

Bacteria and microeukaryotes exhibited similar distribution patterns. Sample clustering by regions revealed significant distance-decay patterns (Fig. 3A–C, Fig. 4). Significant distance-decay patterns of bacteria and/or microeukaryotes have been found in rivers (12, 13), lakes (9, 14), intertidal zones (15), coastal seas (8, 16), and open oceans (16, 17). The underling mechanisms responsible for distance-decay patterns have been shown to differ between domains and between ecosystems (8, 13, 15). The fact that environmental factors varied spatially in the TNPO during our study (Fig. 1B, Fig. S2 and S4) may have led to distinct variations in bacterial and microeukaryotic community structures between the WTNP and CTNP.

Though we found significant correlations between the beta diversities of bacterial and eukaryotic communities for all groups (Fig. S8), the rates of distance-decay differed significantly between domains and/or regions (Fig. 4). We hypothesized that the factors which shaped and the mechanisms which created these beta-diversity patterns differed between domains and regions. Wei et al. (26) have shown that similar drivers could have different effects and hence result in distinct patterns of the beta diversity for soil bacteria and archaea. In our study, environmental variables differed between the WTNP and CTNP, and the relative importance of spatial and environmental factors differed in explaining the differences in community structure between domains and regions (Fig. 1A and 5B and Fig. S1 and S2). Liu et al. (14) have found that different assembly mechanisms govern the similar distance-decay patterns of bacterial and microeukaryotic communities in Tibetan lakes. In lakes located in East Antarctica, the beta-diversity patterns of bacteria and microeukaryotes have been structured by contrasting processes and have been found to differ (9). Previous studies have suggested that the turnover rates of distance-decay patterns are affected by processes which include selection, dispersal limitation, and ecological drift (8, 11). Our study showed that the relative contributions of ecological processes varied between domains and regions (Fig. 5C).

### Assembly mechanisms regulating community structures of microbiota.

In the context of the analytical framework proposed by Stegen et al. (20), our results showed that the relative contributions of ecological processes differed between domains and regions. Ecological drift was the dominant mechanism that explained the assembly of bacterial communities. Heterogeneous selection, dispersal limitation, and ecological drift collectively explained much of the turnover of the microeukaryotic community (Fig. 5C). Consideration of the mechanisms that shaped the surface ocean prokaryote and picoeukaryote communities sampled at 120 tropical and subtropical stations during the Malaspina 2010 voyage indicated that dispersal limitation was the dominant factor shaping the picoeukaryote community, whereas ecological drift, dispersal limitation, and homogeneous selection collectively contributed to turnover of the prokaryotic community (7). Our results were therefore distinctly different from those of Logares et al. (7). Moreover, habitat filtering (species sorting) has been shown to be the dominant mechanism that determines the structure of epipelagic (0 to 200 m) and mesopelagic (200 to 2,000 m) bacterioplankton communities in the Clarion-Clipperton Zone and the Tara Oceans transect (27). One possible explanation for these discrepancies is that the samples used in those studies came from different ecosystems which might have been structured by different mechanisms associated with different biogeochemical conditions and community structures. Specifically, oligotrophic, tropical surface waters were the focus of this study, whereas both the Malaspina and Tara Oceans expeditions included stations distributed among the Longhurst Provinces that differed greatly with respect to environmental factors (7, 27). For example, the temperature ranged from 27.1 to 30.9°C in this study, but it varied from 15.7 to 29.3°C during the study of Logares et al. (7) and from 12.8 to 27.6°C during the study of Lindh et al. (27). In addition, methodological differences may have contributed to the discrepancies between the relative importance of different ecological processes. Previous studies have revealed that amplicon sequencing issues such as primer choices (targeting different variable regions), reference databases for taxonomic assignments, and bioinformatics processing pipelines (e.g., clustering methods [i.e., OTUs, zero-radius OTUs, and amplicon sequence variants], taxonomic classification methods, and specific adjustments [e.g., amplicon truncation]) could have significant influences on the resulting microbial profiles (22) and thus lead to differences between the contributions of different ecological processes. Logares et al. (7) have assessed the impact of sequence clustering (i.e., OTUs based on sequence clustering and sequence variants) on the identification of ecological mechanisms; they found that sequence clustering did not change the main trends, but it could affect measurements of the relative contributions of ecological processes. An alternative explanation for the observed discrepancies in assembly mechanisms between studies may be differences of spatial scale, which have been found to have an important influence on the assembly of microbial communities (21, 28, 29). The spatial distance between sites has been thought to affect ecological mechanisms through its influence on the likelihood of mass effects versus dispersal limitation, biotic interactions, and species sorting (21). However, knowledge of how assembly processes operate in different spatial scales is limited. In our study, the distribution of microeukaryotes was more governed by selection (mainly heterogeneous selection) and dispersal limitation, and less influenced by ecological drift, than was the distribution of bacteria (Fig. 5C). Our results are in accordance with the discovery by Logares et al. (7) that heterogeneous selection is more important in shaping picoeukaryotic than prokaryotic communities. They have speculated that the difference between the effects of selection might be explained by the different modes of adaptation of prokaryotes and picoeukaryotes (7). Microeukaryote cells are larger than bacterial cells, and therefore they would likely be more impacted by dispersal limitation than bacteria (30). Our results indicated that dispersal limitation made a relatively large contribution to the impact of ecological processes on the distribution of microeukaryotes. Both Wu et al. (8) and Logares et al. (7) have noted that differences between dormancy strategies may contribute to differences between the dispersal of bacteria and microeukaryotes because dormancy strategies are more commonly observed in bacteria. Previous studies have assumed that communities with relatively large population sizes would be less influenced by random births and deaths and thus less impacted by ecological drift (8, 31). In this study, we found significantly higher alpha diversity for microeukaryotes relative to bacteria and in the CTNP relative to in the WTNP region. We also found that ecological drift was higher for bacteria than for microeukaryotes and higher in the WTNP than in the CTNP (Fig. 2 and 5C). These results might indicate that ecological drift has more influence on communities with less alpha diversity.

## CONCLUSIONS

Our results indicated that the distribution patterns and factors that influence the alpha and beta diversities of bacteria and microeukaryotes in the oligotrophic waters of the TNPO are similar. Both domains showed significant distance-decay patterns and were clustered according to geographic origin (i.e., the WTNP and CTNP). Spatial variables and environmental factors such as salinity, temperature, and DO were identified as the factors that correlated with both bacterial and microeukaryotic community compositions across the TNPO. The fact that different regions harbored distinct bacterial and microeukaryotic communities showed that it was necessary to consider the effects of environmental heterogeneity and spatial scales in explaining the distributions of microbiota in these waters. Our results also revealed that selection, dispersal, and ecological drift made different contributions in structuring the main components of the marine microbiota. Ecological drift was the principal mechanism responsible for the structure of bacterial communities; heterogeneous selection, dispersal limitation, and ecological drift collectively explained much of the turnover of microeukaryotes. This analysis highlighted the importance of including different taxonomic groups to fully understand the structure, distribution patterns, and assembly mechanisms of marine microbial communities. Future studies focusing on ocean microbiota should investigate how/why the relative importance of selection, dispersal, and ecological drift changes with spatiotemporal scales, nutrient concentrations, and taxonomic groups. Such studies would enhance understanding of the mechanisms that structure marine microbial communities and enable more informed stewardship of marine microbiota in a changing world.

## MATERIALS AND METHODS

### Measurement and processing of environmental variables.

**FIG 6:**
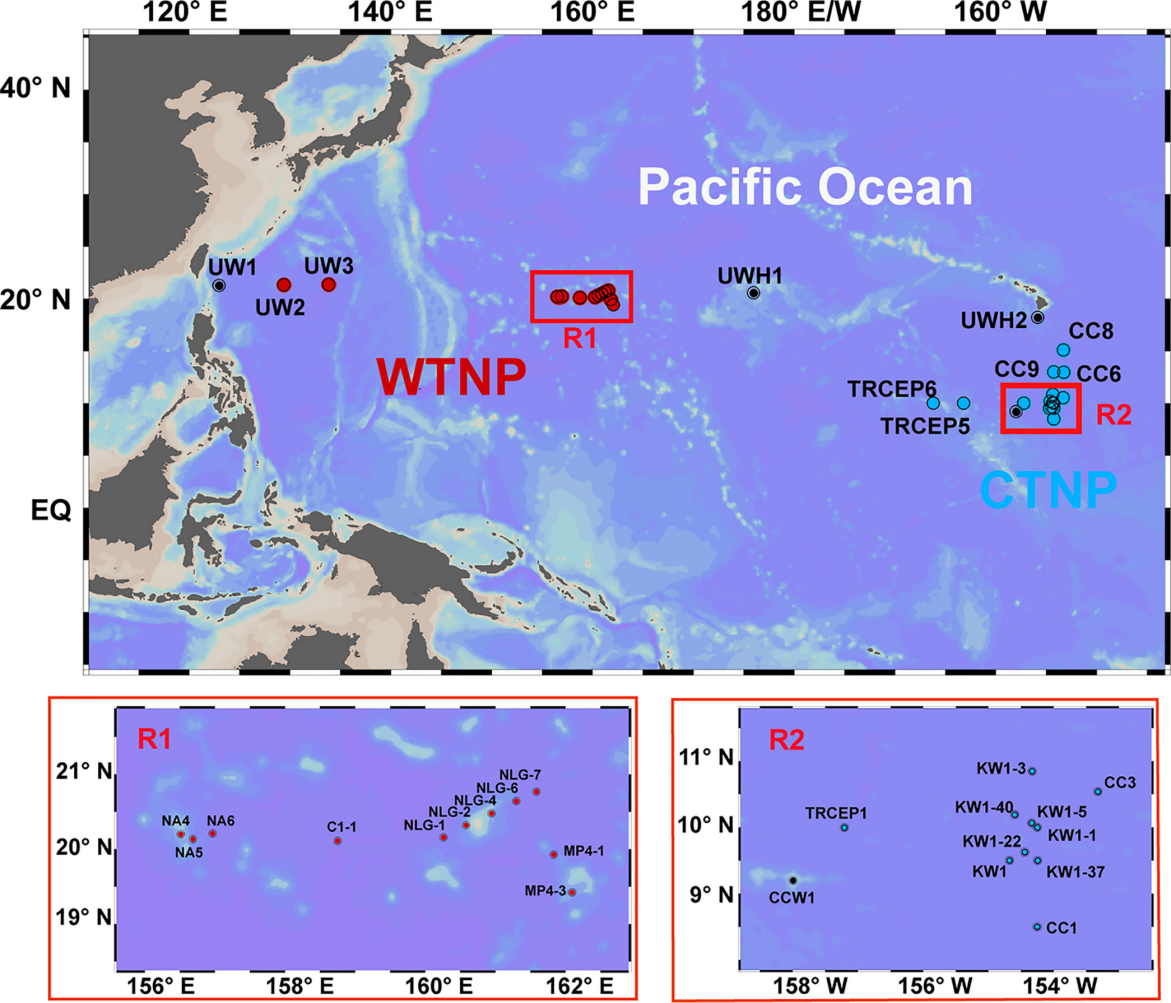
Sampling locations in the tropical North Pacific Ocean. Surface seawater samples were collected from 32 stations. Red dots, the WTNP cluster (13 stations); blue dots, the CTNP cluster (16 stations); black dots, the NA group (3 stations). Table S1 provides detailed information about the stations. The map with sampling locations was constructed using Ocean Data View version 4.7.10.

Sampling was conducted at 32 stations across the western and central TNPO from 18 July to 23 October 2017 as a part of the Dayang 45 cruise (Fig. 6 and Table S1). At each station, a surface sample was collected with Niskin bottles at a depth of 5 m or manually with a plastic bucket from just below the surface. The temperature and salinity of surface seawater were measured using an onboard automatic observing system (SeaBird Electronics SBE45). The pH was measured with a desktop pH meter (Thermo Scientific, USA). The oxidation-reduction potential (Eh) was measured with a desktop Eh meter (Thermo Scientific, USA). Dissolved oxygen (DO) concentrations were determined by the iodometric titration method. Total suspended particulates (TSP) were collected by filtering 4.7 L seawater through preweighed acetate membrane filters (0.45-μm pore size; Millipore, USA). TSP filters were stored at −20°C until processing on shore. The TSP filters were freeze-dried and their weights measured gravimetrically (32). Concentrations of silicic acid (Si(OH)_4_) were determined using the silicon-molybdenum blue method (33). For determination of chlorophyll *a* (Chl *a*) concentrations, we filtered 2.4 L seawater through a GF/F filter (25-mm diameter), which was then stored at −80°C. After extraction of Chl *a* with acetone, Chl *a* concentrations were determined using a Turner Trilogy fluorometer (34). Total organic carbon (TOC) was measured with a TN/TOC analyzer (Shimadzu, Japan). The DO, silicic acid concentrations, and TOC concentrations were determined according to the Specification for Marine Monitoring, Part 4: Seawater Analysis (GB17378.4-2007).

### Analyses of bacterial and microeukaryotic communities.

Microbial cells were collected by filtering 23 L surface seawater (≤5 m) from each station through a 0.2-μm pore-size Isopore membrane filter (142-mm diameter, Merck Millipore, USA) with a peristaltic pump. The filters were kept at −80°C prior to extraction of DNA.

To extract the total genomic DNA, we used the DNeasy PowerWater kit (Qiagen, Germany) and followed the manufacturer’s instructions. To evaluate DNA quality and purity, we used both gel electrophoresis and a NanoDrop 2000 analyzer (Thermo Fisher Scientific, USA). An Invitrogen Qubit 3.0 Spectrophotometer (Thermo Fisher Scientific, USA) was used to accurately measure the DNA concentrations. The hypervariable V3-V4 region of the bacterial 16S rRNA gene was amplified with a forward primer 341F (CCTACGGGNGGCWGCAG) and a reverse primer 805R (GACTACHVGGGTATCTAATCC) (35); the hypervariable V4 of the eukaryotic 18S rRNA gene was amplified with a forward primer TAReuk454FWD1 (CCAGCASCYGCGGTAATTCC) and a reverse primer TAReukREV3 (ACTTTCGTTCTTGATYRA) (36). For each sample, 25-cycle polymerase chain reactions (PCRs) were applied in triplicate. The PCR products were checked via gel electrophoresis and further purified with a commercial kit (Agencourt AMpure XP PCR Purification Beads, Beckman Coulter, USA). Paired-end sequencing was applied with an Illumina Miseq Benchtop Sequencer system.

### Processing and analysis of sequencing data.

Sequencing data were processed and analyzed mainly with USEARCH v10 (37), VSEARCH v2.7.0 (38), and QIIME v.1.9.0 (39). The same pipeline was applied for both bacterial 16S and eukaryotic 18S rRNA gene sequences. Adapter and low-quality (quality scores <20) base pairs were identified and trimmed from the ends of raw paired-end reads using Trim Galore v0.4.5, followed by removal of short reads (<100 bp). Paired-end reads were merged using USEARCH, primers were identified and cut out, quality filtering was performed to discard low-quality reads with >1 total expected errors, and sequences shorter than 300 bp were discarded. Operational Taxonomic Units (OTUs) were clustered at 97% identity using VSEARCH after removing singletons (i.e., sequences that were present only once); chimeras were detected and filtered based on SILVA 132 (40). The representative sequences of OTUs were blasted against SILVA 132 using assign_taxonomy.py in QIIME v.1.9.0. For analysis of the bacterial community, an OTU table was randomly rarefied to 95,250 sequences after nonbacterial OTUs such as archaea and chloroplast OTUs had been filtered out. For microeukaryotic community analysis, an OTU table was randomly rarefied to the same depth as the bacterial OTU table after non-microeukaryotic OTUs (e.g., metazoa) had been filtered out. Indices of alpha diversity (species richness, Faith’s phylogenetic diversity [PD; 41], and the Shannon diversity index) were estimated with the command alpha_diversity.py in QIIME.

### Statistical analyses.

All statistical analyses as well as the production of figures were carried out with R project (version 3.6.1; 42) unless otherwise noted. The nearest taxon index (NTI; 43), which was used as a phylogenetic metric to quantify the extant of phylogenetic relatedness among species within a community, was calculated with 999 randomizations using the R package picante (44). Mean value comparisons were performed using a Wilcoxon signed-rank test. A principal-component analysis (PCA) was performed on the Z-score standardized environmental variables. Principal coordinates analysis (PCoA) was applied to reveal distribution patterns of bacterial and microeukaryotic communities based on the Bray-Curtis dissimilarity matrix. Analysis of similarity (ANOSIM) with 9,999 permutations was used to further statistically assess between-group differences. To test the agreement of distribution patterns between bacteria and microeukaryotes, we used Procrustes analysis based on the PCoA results; the statistical significance was assessed with a Monte Carlo analysis. Spearman correlation coefficients were calculated between the alpha diversities of bacteria and microeukaryotes and measured environmental variables. The type I error rates (*P* values) of measured environmental variables were adjusted via the “fdr” method. The function diffslope() in the R package simba was used to calculate the difference in slopes of regression lines (45). Mantel tests were used to find factors that were significantly correlated with variations of bacterial and/or microeukaryotic abundance. The PCA, PCoA, ANOSIM, Procrustes, and Mantel test analyses were all performed using the R package vegan (46).

We applied the phylogenetic null model (20) to elucidate the mechanisms that shaped the communities of microbiota. According to the analytical framework proposed by Stegen et al. (20), the relative contributions of ecological processes such as selection, dispersal, and ecological drift could be estimated based on the phylogenetic null model. The estimation involves the following two major steps. The first step is to determine the contribution of selection by estimating the beta Nearest Taxon Index (βNTI) using the phylogenetic turnover between communities. The second step is to estimate the influences of dispersal and ecological drift by calculating the Bray-Curtis-based Raup-Crich (RC_bray_) using the OTU composition turnover (20).

### Data availability.

All raw data were submitted to the National Center for Biotechnology Information Sequence Read Archive (accession number PRJNA733026 and PRJNA733279 for 16S and 18S rRNA gene sequences, respectively).
