# Supplementary material for: Contrasting Community Assembly Mechanisms Underlie Similar Biogeographic Patterns of Surface Microbiota in the Tropical North Pacific Ocean
Source: Microbiol Spectr. 2022 Jan 12;10(1):e00798-21. doi: 10.1128/spectrum.00798-21 (PMC8754141; doi:10.1128/spectrum.00798-21)
Supplement: SUPPLEMENTAL FILE 1 — Supplemental material. Download SPECTRUM00798-21_Supp_1_seq9.pdf, PDF file, 1.0 MB [file spectrum00798-21_supp_1_seq9.pdf]

Supporting tables and figures

Table S1 |Environmental factors for the 32 surface seawater samples in the TNPO. Name, sample names used in this study; N_id, sample names used for NCBI submission; Date, day/month/year; Lon, longitude of sampling location; Lat, latitude of sampling location; Depth, bottom depth of sampling station; T, temperature; DO, dissolved oxygen; Eh, oxidation-reduction potential; TSP, total suspended particulate; TOC, total organic carbon. According to locations and values of temperature and salinity, samples were classed into WTNP (high temperature and salinity) and CTNP (low temperature and salinity) regions. NA means data not available. Sample names in bold indicate that surface seawater was sampled manually.

Name	N_id	Date	Lon	Lat	Depth m	T ℃	Salinity psu	DO µmol/L	Eh mv	pH	TSP mg/L	$Si(OH)_4$ µmol/L	Chl *a* µg/L	TOC mg/L	Group
**UW1**	(euk)DY28	18/7/2017	123.00	21.28	NA	NA	NA	NA	NA	NA	NA	NA	0.036	NA	NA
**UW2**	(euk)DY29	19/7/2017	129.42	21.32	NA	30.91	34.48	NA	NA	NA	NA	NA	0.111	NA	WTNP
**UW3**	(euk)DY30	20/7/2017	133.86	21.36	NA	30.67	34.73	NA	NA	NA	NA	NA	0.112	NA	WTNP
NA4	(euk)DY17	24/7/2017	156.51	20.20	1725	30.23	34.62	386.8	437.2	8.31	4.5	0.960	0.091	1.05	WTNP
NA5	(euk)DY18	26/7/2017	156.69	20.14	2312	29.88	34.78	390.4	238.6	8.18	7.0	0.425	0.115	0.87	WTNP
NA6	(euk)DY19	28/7/2017	156.97	20.21	5168	29.86	34.91	389.1	48.3	8.13	2.8	0.728	0.099	0.87	WTNP
C1-1	(euk)DY01	29/7/2017	158.75	20.11	5517	29.46	34.95	388.0	116.1	8.14	5.1	0.611	0.133	0.91	WTNP
NLG1	(euk)DY20	31/7/2017	160.26	20.16	4937	29.58	35.27	393.4	287.0	8.21	1.9	0.774	0.083	1.07	WTNP
NLG2	(euk)DY21	01/8/2017	160.58	20.32	2016	29.62	35.15	390.4	270.1	8.15	6.4	0.960	0.089	0.96	WTNP
NLG4	(euk)DY22	02/8/2017	160.95	20.48	2618	29.36	34.96	388.4	312.9	8.17	1.0	0.588	0.095	0.69	WTNP
NLG6	(euk)DY23	02/8/2017	161.30	20.64	4361	29.40	35.18	392.5	303.2	8.22	11.8	0.356	0.282	0.95	WTNP
NLG7	(euk)DY24	03/8/2017	161.59	20.77	5197	29.53	35.19	389.6	193.0	8.19	2.7	1.61	0.133	0.92	WTNP

MP4-1	(euk)DY15	05/8/2017	161.83	19.93	4338	29.52	34.91	393.6	267.4	8.21	0.9	0.542	0.097	0.63	WTNP
MP4-3	(euk)DY16	06/8/2017	162.10	19.42	2912	29.75	34.87	390.0	239.0	8.20	1.9	0.402	0.038	0.97	WTNP
**UWH1**	(euk)DY31	06/8/2017	175.97	20.55	NA	28.44	34.39	NA	NA	NA	NA	NA	NA	NA	CTNP
**UWH2**	(euk)DY32	20/8/2017	-155.85	18.21	NA	NA	NA	NA	NA	NA	NA	NA	NA	NA	NA
CC6	(euk)DY04	21/8/2017	-153.24	12.97	5447	27.68	34.48	400.7	307.4	8.22	1.9	0.138	0.075	1.10	CTNP
**CC8**	(euk)DY05	21/8/2017	-153.27	15.12	5826	27.09	34.49	NA	NA	NA	NA	NA	NA	NA	CTNP
**CC3**	(euk)DY03	24/8/2017	-153.32	10.54	4961	28.71	34.03	NA	NA	NA	NA	NA	NA	NA	CTNP
CC1	(euk)DY02	25/8/2017	-154.25	8.50	5187	28.87	34.12	394.7	362.4	8.24	2.1	0.341	0.075	1.00	CTNP
**KW1**	(euk)DY08	26/8/2017	-154.68	9.50	5229	28.58	34.19	NA	NA	NA	NA	NA	NA	NA	CTNP
**KW1-22**	(euk)DY11	04/9/2017	-154.44	9.63	5198	28.59	34.18	NA	NA	NA	NA	NA	NA	NA	CTNP
KW1-37	(euk)DY12	05/9/2017	-154.25	9.50	5138	28.49	34.16	400.2	354.6	8.25	1.0	4.20	0.115	1.00	CTNP
KW1-5	(euk)DY10	10/9/2017	-154.34	10.07	5175	28.65	34.28	402.5	375.1	8.12	0.6	0.205	0.082	0.46	CTNP
**KW1-3**	(euk)DY09	15/9/2017	-154.33	10.85	5065	28.62	34.15	NA	NA	NA	NA	NA	NA	NA	CTNP
KW1-1	(euk)DY14	17/9/2017	-154.25	10.00	5239	29.20	34.29	398.3	272.4	8.29	3.3	0.634	0.079	1.04	CTNP
KW1-40	(euk)DY13	19/9/2017	-154.59	10.19	5148	28.46	34.05	398.9	265.3	8.33	1.4	0.250	0.093	0.91	CTNP
CC9	(euk)DY06	07/10/2017	-154.26	12.99	5414	28.11	34.07	383.7	267.2	8.24	1.2	0.648	0.093	1.09	CTNP
CCW1	(euk)DY07	16/10/2017	-157.99	9.20	1301	28.45	NA	401.2	321.2	8.18	2.2	0.858	0.161	0.86	NA
TRCEP1	(euk)DY25	18/10/2017	-157.21	10.00	5346	28.68	33.80	402.7	329.3	8.18	2.3	0.975	0.211	0.92	CTNP
TRCEP5	(euk)DY26	21/10/2017	-163.20	10.01	4060	28.86	34.17	400.9	317.0	8.17	2.6	0.812	0.127	0.89	CTNP
TRCEP6	(euk)DY27	23/10/2017	-166.20	10.00	4457	28.48	33.82	400.3	285.8	8.19	1.5	1.05	0.236	0.87	CTNP

Table S2 | Alpha diversity indices for the 32 bacterial and microeukaryotic communities in the TNPO. HQR: the number of high quality reads assigned to bacteria or microeukaryotes; Richness: observed number of OTUs; PD: Faith's phylogenetic diversity; NTI: nearest taxon index. For calculation of the NTI, the number of randomizations was 999. *P* values <0.05 are in bold.

Name	Bacteria					Microeukaryotes				
	HQR	Richness	PD	Shannon	NTI	HQR	Richness	PD	Shannon	NTI
C1-1	136883	510	47.2	4.98	**2.63**	166459	1558	120.2	7.34	**3.39**
CC-1	130507	613	51.0	5.30	**2.47**	140961	1790	137.9	7.75	**3.51**
CC-3	170502	640	55.3	5.54	**2.46**	225216	1661	128.5	7.39	**2.30**
CC-6	126569	665	53.0	5.77	**2.40**	152783	1801	140.2	7.60	**3.11**
CC-8	117845	652	53.9	5.44	**2.20**	224402	1989	146.8	8.17	**3.92**
CC-9	127725	862	69.5	5.72	**2.14**	132263	1897	149.2	7.91	**3.60**
CCW-1	131093	836	66.4	5.66	**2.59**	177933	1745	150.2	7.72	**3.19**
KW1	132599	580	51.6	5.54	**2.55**	227587	1559	122.2	7.38	**3.04**
KW1-1	147764	931	71.6	6.16	**2.51**	187978	1681	136.7	7.39	**2.88**
KW1-3	121726	887	69.9	5.56	**2.43**	145020	1863	150.1	7.60	**2.51**
KW1-22	146798	822	64.2	5.79	**2.78**	166632	1481	123.3	6.49	**2.70**
KW1-37	127755	776	62.9	5.79	**2.45**	187104	1819	139.8	7.72	**3.48**
KW1-40	113547	773	60.1	5.65	**2.44**	160997	1727	138.6	7.40	**3.01**
KW1-5	131259	745	59.9	5.49	**2.47**	226757	1880	148.9	7.85	**2.62**
MP4-1	146186	552	49.9	5.59	**2.96**	151767	1587	117.9	7.31	**3.49**
MP4-3	105681	506	44.8	5.56	**3.11**	115210	1421	112.1	6.99	**3.06**
NA-4	110438	516	49.2	4.99	**2.44**	114528	1292	104.4	6.97	**3.64**
NA-5	147398	568	52.1	5.11	**2.53**	129082	1727	135.3	7.53	**3.42**
NA-6	123041	622	55.7	5.08	**2.21**	193628	1701	134.1	7.27	**2.75**
NLG-1	134825	544	48.7	5.34	**2.87**	171462	1510	121.8	6.81	1.12
NLG-2	163023	606	53.9	5.38	**2.70**	169873	1554	127.5	7.15	**1.72**
NLG-4	106268	505	48.1	4.99	**2.94**	171797	1475	123.0	7.05	**2.69**
NLG-6	120603	500	46.3	5.07	**3.00**	177801	1598	129.3	7.25	**3.25**
NLG-7	161342	572	51.0	5.22	**3.07**	242497	1745	140.0	7.68	**3.60**
TRCEP1	123713	1150	85.4	5.86	**2.50**	202306	2148	178.4	8.01	**3.20**
TRCEP5	125166	853	69.4	5.78	**2.64**	246021	1975	150.9	7.95	**3.02**
TRCEP6	155040	952	78.0	5.70	**2.64**	168036	1687	147.9	7.41	**3.30**
UW1	129275	418	40.8	5.23	**2.79**	207805	1638	124.1	7.60	**3.58**
UW2	161620	381	37.4	5.29	**2.81**	191023	1408	107.5	7.15	**3.11**
UW3	95250	369	35.8	5.16	**2.65**	223290	1502	113.1	7.50	**3.21**
UWH1	139289	646	54.3	5.58	**2.50**	214628	1554	123.2	6.67	-0.86
UWH2	140568	735	59.9	5.75	**2.37**	137795	1893	140.1	7.89	**3.80**

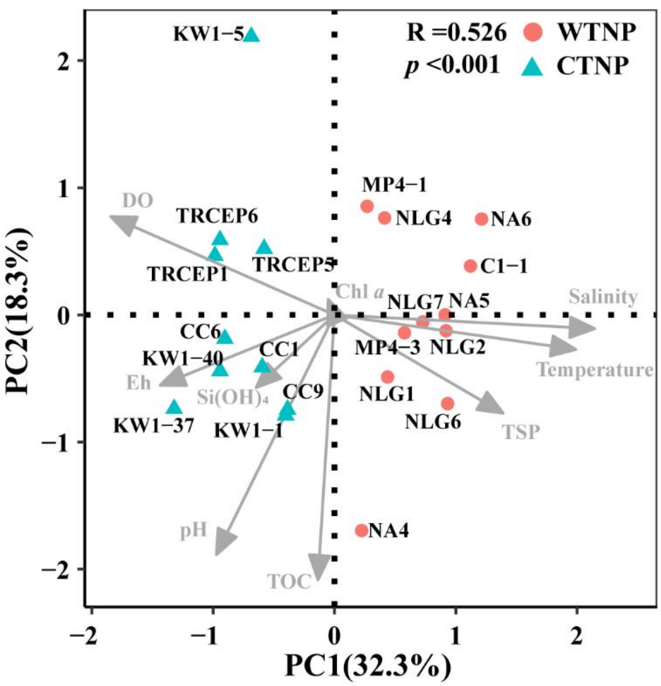

Figure S1 | Principal component analysis of environmental factors. All environmental variables were Z-score standardized and used to calculate the Euclidean distance. The R and *p* statistics are the results of ANOSIM analysis with 9999 permutations. This analysis was used to further assess between-group difference statistically.

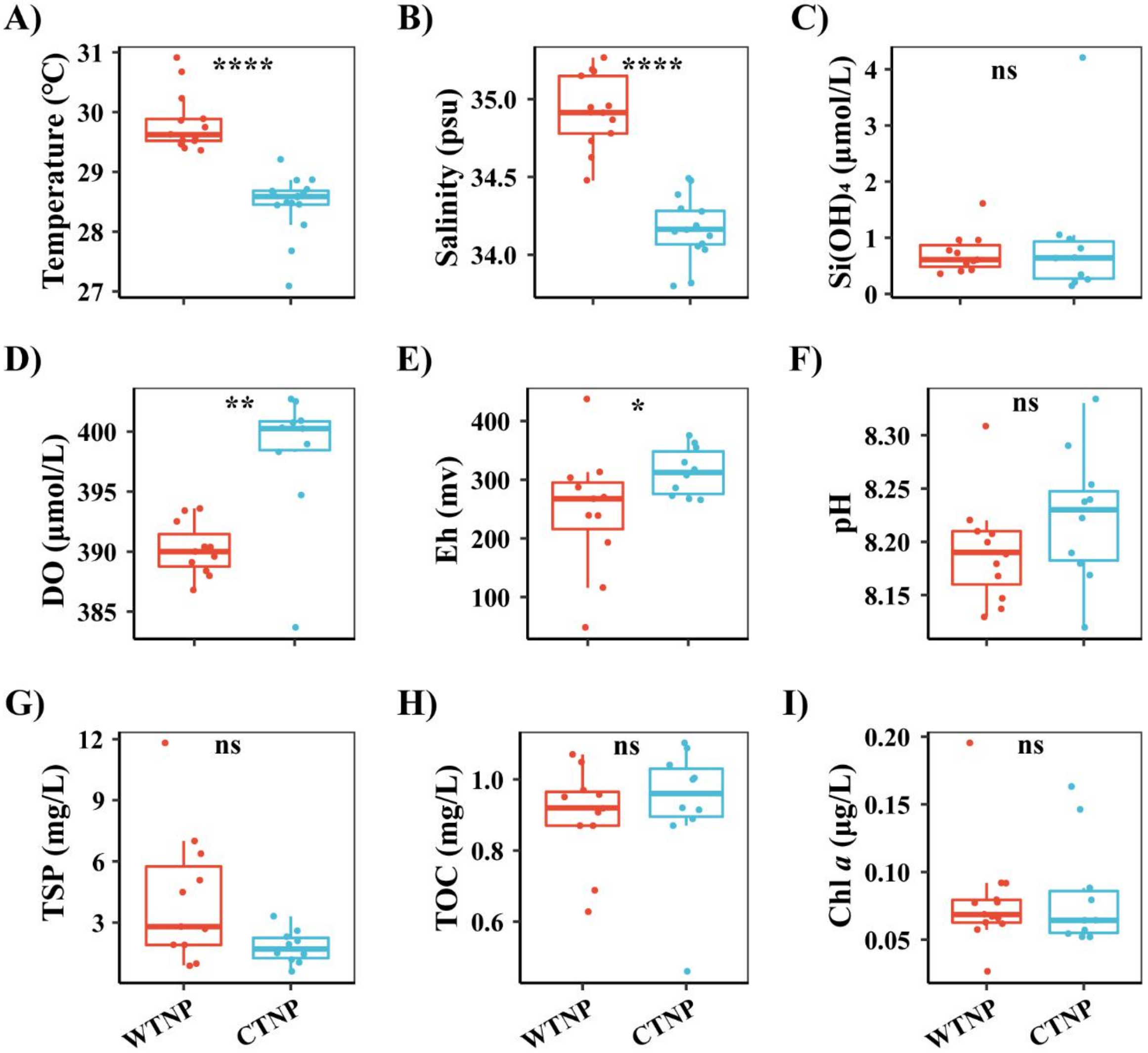

Figure S2 | Boxplot comparing mean values of environmental variables, i.e., **A)** temperature, **B)** salinity, **C)** $Si(OH)_4$ concentrations, **D)** DO, **E)** Eh, **F)** pH, **G)** TSP, **H)** TOC and **I)** Chl *a* concentration. Mean value comparisons were performed using a Wilcoxon test. **** means *p* value ≤0.0001; ns, *p* value >0.05.

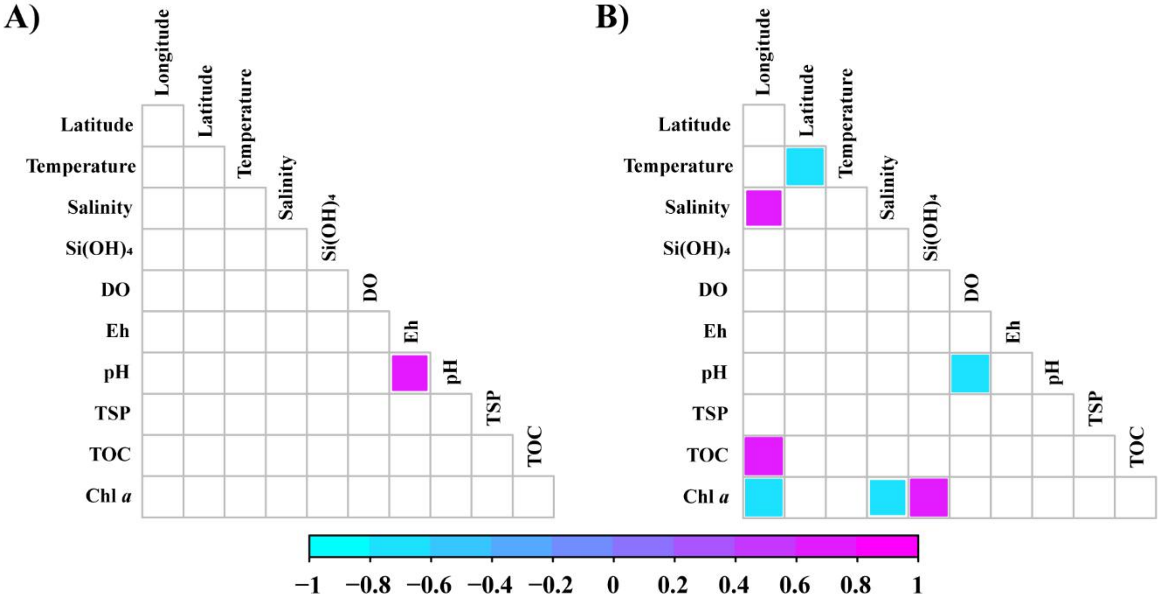

Figure S3 | Spearman correlation matrix showing the results of Spearman rank correlation tests among spatial and environmental factors for the **A)** WTNP and **B)** CTNP. The *p* values were adjusted with the "fdr" method. Only adjusted *p* values <0.05 are shown.

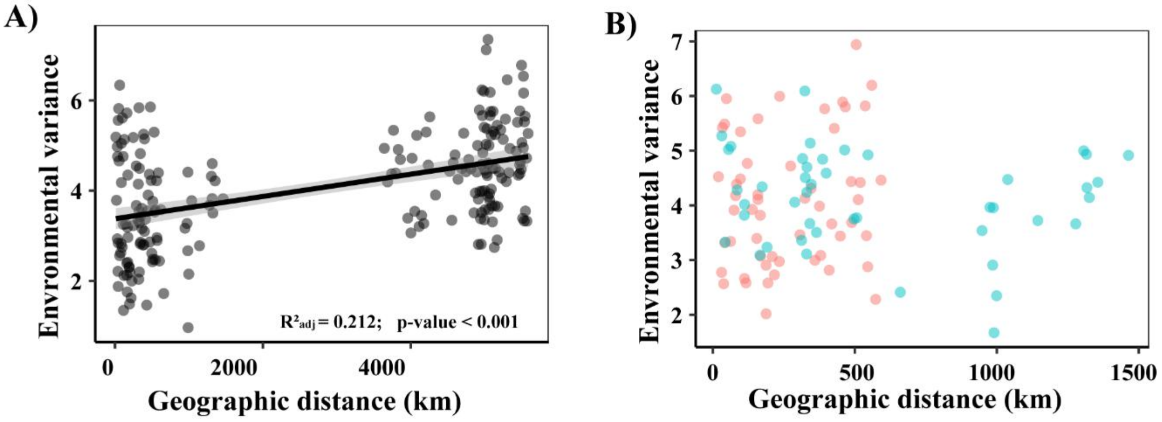

Figure S4 | Pairwise environmental variances as functions of geographic distances. Environmental variables were Z-score standardized and used to calculate the Euclidean distance. **A)** all samples from the TNPO, **B)** samples from within the WTNP and CTNP regions. Only significant regressions are shown with lines. The solid line shows the best linear fit.

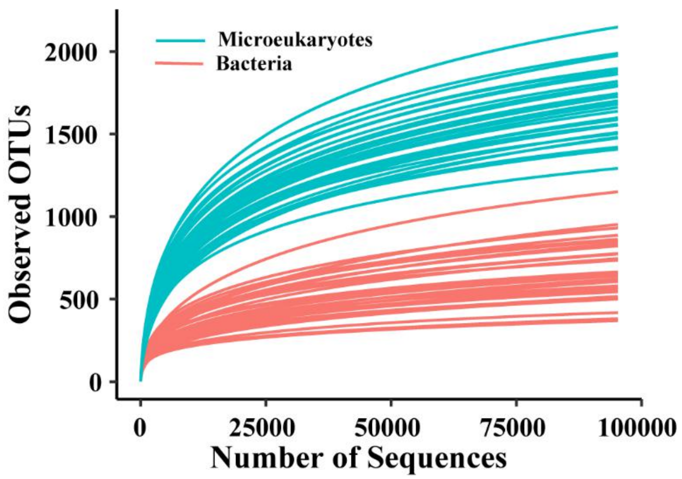

Figure S5 | Rarefaction curves of similarity-based operational taxonomic units at 97% similarity.

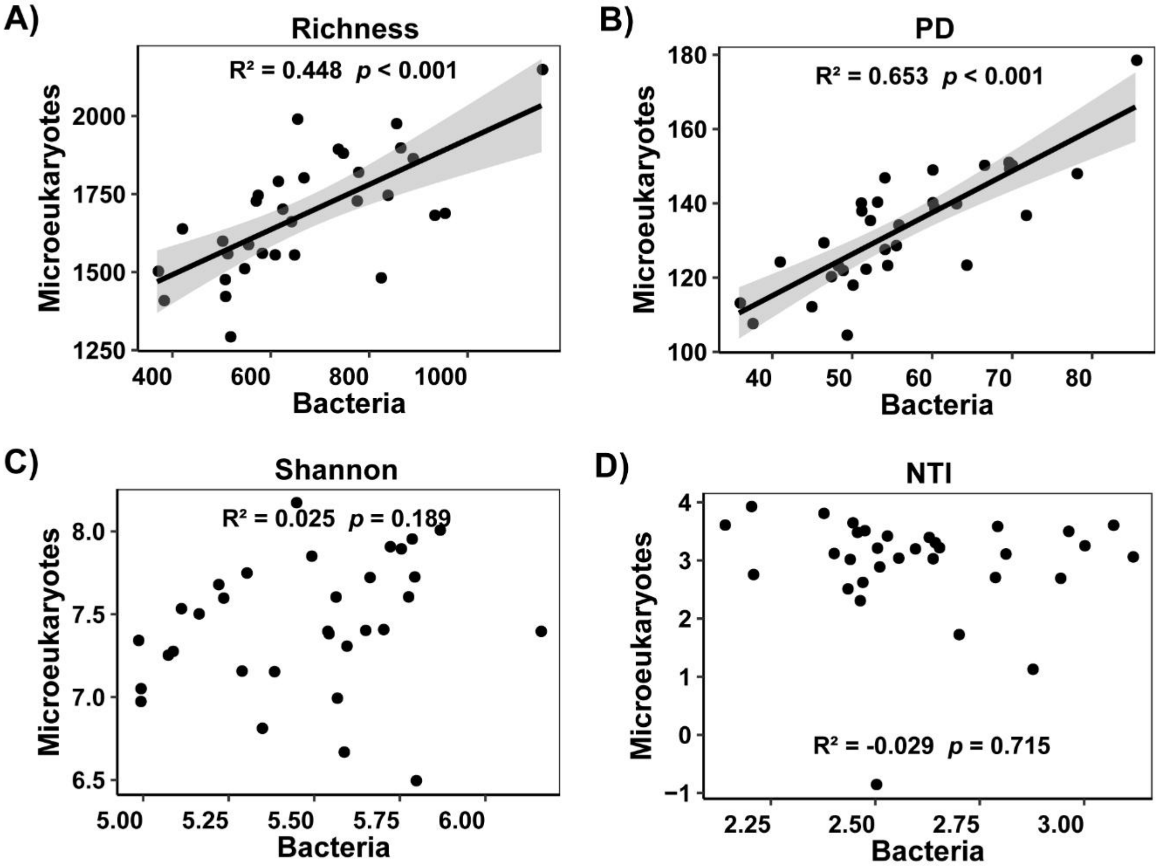

Figure S6 | Correlations for **A)** Species richness, **B)** PD, **C)** Shannon diversity, **D)** NTI of total bacterial and microeukaryotic communities. Only significant regressions are shown with lines. Solid lines show the best linear fit.

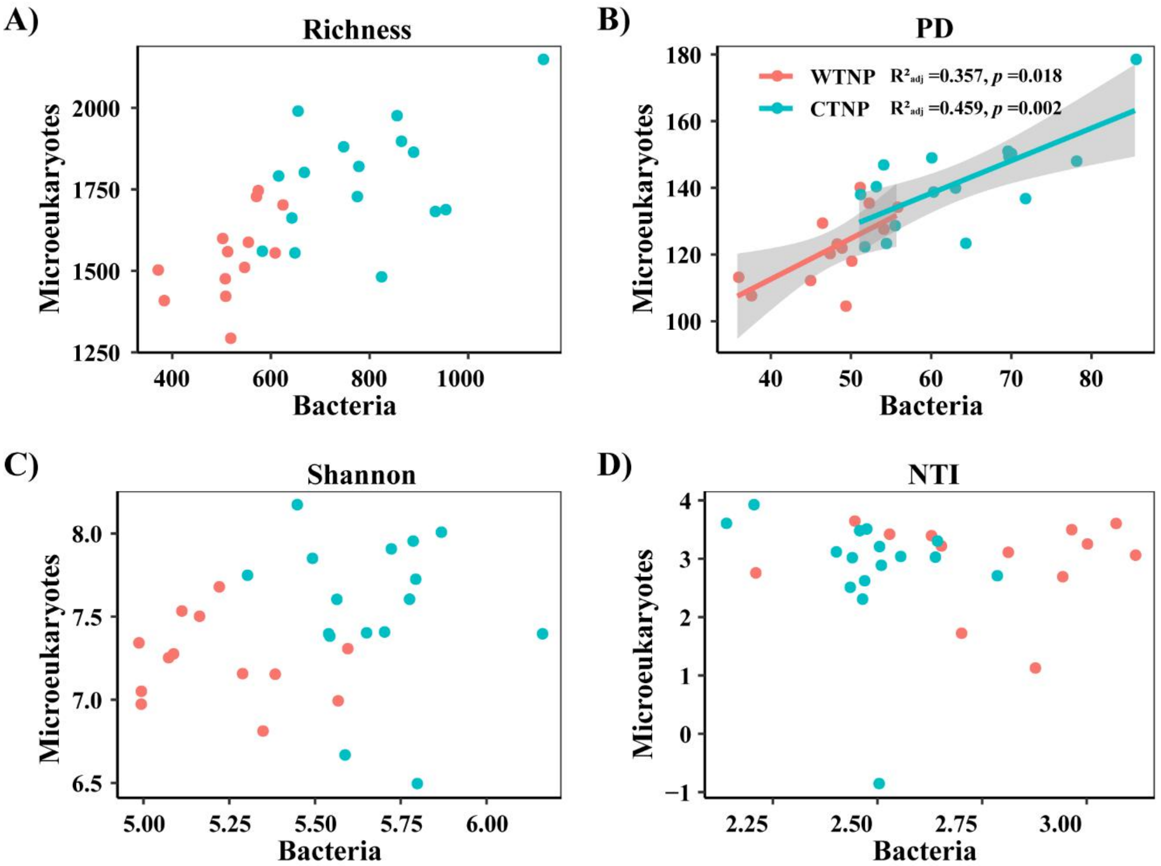

Figure S7 | Correlations for **A)** Species richness, **B)** PD, **C)** Shannon diversity, **D)** NTI of bacterial and microeukaryotic communities in the WTNP and CTNP. Only significant regressions are shown with lines. The solid line shows the best linear fit.

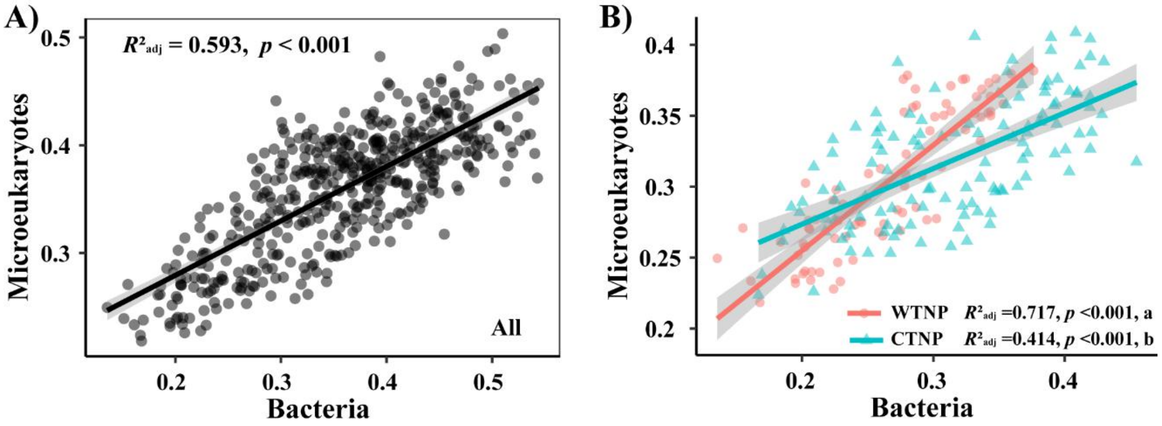

Figure S8 | Bacterial versus microeukaryotic beta diversity (Bray-Curtis distances) for **A)** TNPO communities, and **B)** WTNP and CTNP communities, separately. Solid lines show the best linear fits.

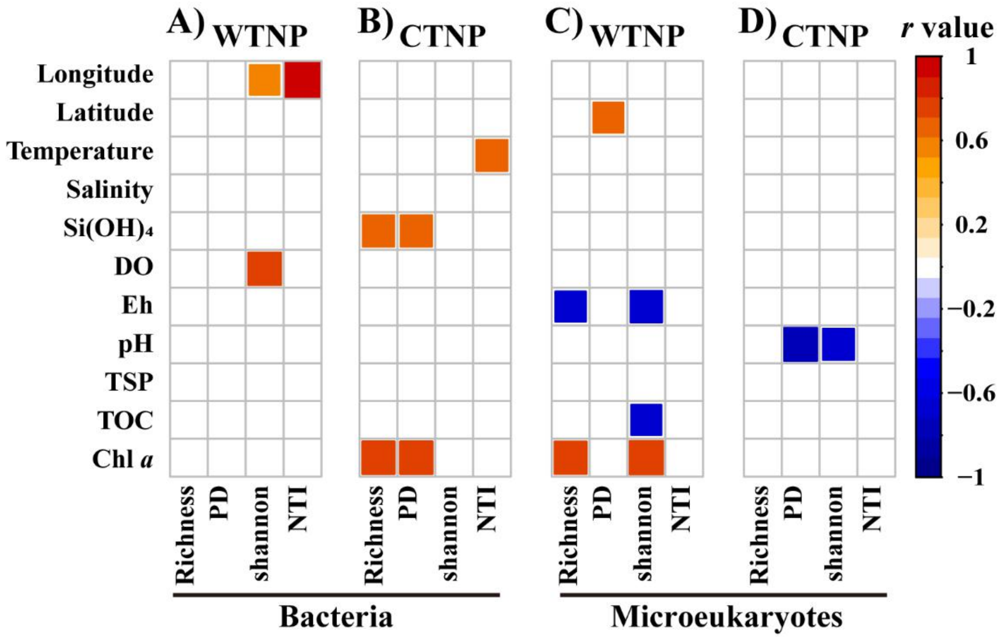

Figure S9 | Heatmaps showing the results of Spearman rank correlation tests between spatial and environmental factors and alpha diversity indices of **A)** WTNP bacteria, **B)** CTNP bacteria, **C)** WTNP microeukaryotes, and **D)** CTNP microeukaryotes. The *p* values were adjusted with “fdr” method. Only adjusted *p* values <0.05 are shown.
